# Persistence-Weighted Performance Metric for PID Gain Optimization in Optical Tracking of Unknown Space Objects

**DOI:** 10.3390/s25216659

**Published:** 2025-11-01

**Authors:** Chul Hyun, Donggeon Kim, Hyunseung Kim, Seungwook Park

**Affiliations:** LIG Nex1, Seongnam-si 13488, Republic of Korea; donggeon.kim@lignex1.com (D.K.); hyunseung.kim2@lignex1.com (H.K.); seungwook.park@lignex1.com (S.P.)

**Keywords:** optical tracking, space object identification, PID control, genetic algorithm, performance metric, offset correction, tracking stability, PWTI

## Abstract

Optical tracking of unknown space objects requires both spatial accuracy and temporal stability to enable high-resolution identification through narrow field-of-view sensors. Traditional performance indices such as RMS error and persistence time (PT) have been used for controller tuning, but they each capture only a subset of the requirements for successful optical identification. This paper proposes a new composite metric, the Persistence-Weighted Tracking Index (PWTI), which combines spatial precision and segment-level continuity into a single measure. The metric assigns a frame-level score based on positional error and accumulates weighted scores over the longest continuous in-threshold segment. Using PWTI as the optimization objective, a genetic algorithm (GA) is employed to tune the PID gains of a frame-by-frame offset correction controller. Comparative simulations under various observation scenarios demonstrate that the PWTI-based approach outperforms RMS- and PT-based tuning methods in both alignment accuracy and consistency. The results validate the proposed metric as a more suitable performance indicator for optical identification tasks involving unknown or uncataloged targets.

## 1. Introduction

Efforts to enhance space domain awareness (SDA) have led to the development of various observation and tracking systems. Among these, optical observation serves as a passive alternative to radar and active laser-based technologies, especially in situations where cooperative tracking or onboard transponders are not available. Although optical systems are inherently constrained by lighting conditions and limited observation windows, they remain a practical option for monitoring non-cooperative targets under favorable environmental conditions.

A typical optical tracking configuration employs a wide field search telescope for initial detection and orbit estimation, followed by an identification telescope with a narrow field of view to capture high resolution images. When dealing with unknown or unregistered space objects, no prior orbital data are available, and orbit estimation must rely on observations collected over a limited portion of the orbit. This process introduces significant uncertainty in trajectory prediction, which in turn makes it challenging to maintain the object near the center of the field of view during the identification phase.

In this study, we implemented a PID controller that corrects positional offsets between predicted and observed target locations on a per-frame basis. Although the control structure follows a conventional PID form, the operational context diverges significantly from typical scenarios with fixed reference trajectories. In our case the reference is derived from initial orbit determination (IOD) based on a limited number of optical measurements. As a result it tends to deviate from the actual target motion in ways that are highly dependent on the observation site, IOD timing and target geometry. These deviations are time-varying and often difficult to predict, making it challenging to define a stable or consistent control objective. Consequently, offset correction must respond not only to momentary tracking errors but also to the evolving discrepancy between the estimated and true trajectories, rendering traditional tuning approaches such as frequency-domain analysis or model-based techniques less effective.

Previous attempts to tune PID gains for this application have utilized grid search [1] and genetic algorithms (GA), using performance metrics such as RMS error [2] and persistence time [3]. These indicators capture average error magnitude and the total time spent within a defined error threshold, respectively. However, they do not fully represent the combined requirement of precise alignment and consistent tracking, both of which are essential for successful optical identification.

To address this challenge, we propose the Persistence-Weighted Tracking Index (PWTI), which integrates spatial accuracy and temporal stability within a single quantitative metric. Using PWTI as the objective, a genetic-algorithm-based PID tuning process is employed so that gain optimization directly reflects realistic tracking behavior. Across multiple observation scenarios, this approach yields a more consistent balance between accuracy and stability than conventional empirical tuning.

The remainder of this paper is organized as follows. Section 2 reviews related work; Section 3 presents the PWTI formulation and the GA-based optimization framework; Section 4 reports the simulation setup and results; Section 5 concludes with limitations and future directions.

## 2. Related Work

### 2.1. Optical Tracking and Identification for Space Objects

Space surveillance involves a variety of sensing modalities, each with its own advantages and limitations. Radar systems are well established for wide-area detection and all-weather tracking, while laser-based techniques are effective for precision ranging under favorable conditions. In parallel, optical observation has been employed as a complementary method, offering the benefits of passive sensing, relatively low infrastructure cost, and high angular resolution, particularly useful for observing objects that do not emit signals or possess cooperative transponders.

Although optical systems are constrained by environmental factors such as lighting and weather, they remain an accessible and practical tool for monitoring space objects, especially when used in coordination with other sensing platforms. Numerous studies have investigated the development of optical tracking architectures and orbit estimation methods for SSA applications, particularly under limited signal conditions [4,5,6]. Optical tracking is especially advantageous when identifying or monitoring previously unknown or uncatalogued objects, where no prior orbital elements are available. In such cases, ground-based telescopes must rely solely on reflected sunlight, often limited to twilight observation windows when illumination and contrast conditions are briefly favorable.

A typical ground-based optical tracking system is structured in two stages: a wide-field telescope first performs initial detection and gathers short-arc observations; these data are used to perform IOD. The result is then handed off to a narrow-field identification telescope, which attempts to reacquire and track the object for high-resolution imaging. This general architecture has also been realized in small telescope or cost-sensitive SSA implementations [7].

This second stage, however, introduces additional challenges due to the extremely small field of view (FOV) of the identification telescope, making accurate alignment critical. In such systems, it is not sufficient for the object to remain within the frame; rather, it must stay within just a few arcseconds of the image center for a sustained period to allow for high-resolution imaging. Even minor errors in predicted trajectory or tracking response can result in the object drifting outside the usable region, compromising image quality or causing the observation to fail entirely.

These constraints demand a control strategy that goes beyond conventional path following. Instead, the system must act as a dynamic regulator that continually adjusts to evolving offset errors and prediction uncertainties. Recent literature has highlighted challenges in daytime tracking [8], faint object detection [9], and sensor design under low signal-to-noise conditions [10], all of which emphasize the need for robust tracking mechanisms.

Our previous work has explored feedback-based correction schemes that use image-derived offset signals. While proportional correction can suffice under moderate conditions, more robust control structures are required when dealing with high-elevation angles or uncertain initial orbit estimates [1]. These conditions motivate the development of dedicated control solutions tailored to identification-focused optical tracking systems.

### 2.2. PID-Based Offset Control in Optical Tracking Systems

Conventional PID gain tuning methods, including frequency-domain analysis, pole placement, and Ziegler–Nichols heuristics, typically assume that the system model is explicitly known and that the reference trajectory remains stable. However, in offset tracking scenarios based on IOD, these assumptions often do not apply. The reference is not fixed but is instead continuously adjusted using measurement values extracted from images, which change over time and are affected by noise and uncertainty. Furthermore, delays in the control loop and limited knowledge of actuator dynamics make analytical tuning either difficult or unreliable.

This difficulty is especially prominent in IOD-based systems, where short-arc observations and range ambiguity lead to high uncertainty in predicted trajectories [11]. In cases where the control reference varies over time and cannot be clearly defined in advance, various metaheuristic optimization methods have been applied for gain tuning. Common examples include Genetic Algorithms (GA), Particle Swarm Optimization (PSO), and Differential Evolution (DE). These techniques do not depend on exact system models or gradient information. Instead, they search over candidate solutions by evaluating how each one performs in simulation, making them well suited for complex and nonlinear systems.

Among these approaches, GA is particularly favored in PID control due to its simple conceptual design and consistent robustness. The algorithm operates by generating a group of candidate gain combinations and improving them through repeated iterations. Each candidate is assessed according to a performance score that reflects the specific control objective. This strategy helps preserve diversity within the search process and reduces the risk of converging too quickly on suboptimal solutions, especially valuable in systems that exhibit unpredictability or nonlinear behavior.

Although PSO and DE are also effective for continuous optimization problems, they often require more careful parameter tuning or may show signs of early convergence in certain applications. In this study, GA was selected due to its ease of use, compatibility with performance evaluations based on simulation, and positive results in previous control experiments related to this architecture.

This empirical tuning method is well aligned with the offset tracking environment considered in this research, where traditional model-based control design is difficult to implement. Evolutionary algorithms allow the tuning process to adapt naturally to practical uncertainties and eliminate the need for precise mathematical modeling of the plant.

### 2.3. Metaheuristic Approaches to PID Gain Tuning

Traditional PID tuning methods such as Ziegler–Nichols heuristics or frequency-based design assume that the system model is well understood and that the reference trajectory remains constant. In offset tracking scenarios where the reference changes over time and accurate system modeling is difficult, alternative tuning strategies are often more effective. Metaheuristic optimization has gained interest in such cases, with numerous studies applying it for PID gain tuning under uncertain and nonlinear dynamics [12,13,14].

GA are widely used for PID tuning due to their flexibility and robustness. GA operates by iteratively updating a group of candidate gain sets through selection, crossover, and mutation. Each candidate is evaluated using a chosen performance metric, and the algorithm explores the solution space over successive generations. Its success in PID applications across diverse systems such as motors, UAVs, and underwater vehicles is well documented [14,15,16].

Another widely used method is particle swarm optimization (PSO), which mimics the collective behavior of particles that adjust their positions based on personal and group performance. PSO can converge more quickly in smooth optimization problems and requires fewer configuration parameters than GA. However, it may show premature convergence or reduced reliability in noisy or complex environments [17].

Differential evolution (DE) has also been applied to continuous parameter tuning. It modifies candidate solutions by combining scaled differences between them and is known for its stable performance in high-dimensional spaces. Still, the algorithm’s performance can be sensitive to the selection of its mutation and crossover rates [18].

Other methods such as simulated annealing (SA) and ant colony optimization (ACO) have been applied to specific control tasks [19,20]. Although SA helps avoid local minima through probabilistic exploration and ACO is strong in discrete problem domains, these methods are less commonly used in PID tuning due to limited applicability.

In this study, we adopt GA as our optimization method due to its previous success in similar tracking scenarios and its compatibility with simulation-based evaluation. Our tracking configuration requires the controller to respond to frame-by-frame positional errors under uncertain dynamics, without direct access to detailed actuator models. GA enables effective gain tuning under these practical constraints.

Prior work on simulation-based PID tuning has also shown that GA performs robustly even when standard control models are unavailable [12,13,15]. Moreover, in our previous work on narrow field-of-view optical tracking systems, GA consistently yielded effective PID gain sets for offset compensation. For these reasons, GA serves as both the tuning mechanism in this study and the shared optimization framework used to evaluate different performance metrics in the sections that follow.

### 2.4. Performance Metrics in Optical Offset Tracking

In previous studies on optical offset tracking, performance evaluation has typically relied on indicators such as RMS error and persistence time. RMS error provides an overall measure of deviation from the reference, while persistence time reflects how long the system remains within a specified error threshold. These metrics have been applied to various controller tuning studies across robotic, aerial, and marine domains [14,15,16,21,22].

However, in optical systems designed for space object identification, the operational requirements differ significantly from general tracking scenarios. The critical objective is not only to keep the target within the field of view but to maintain it stably and precisely aligned near the center of the image over a sustained duration. In such cases, average performance or coarse error bounds may not sufficiently reflect the actual quality of alignment needed for image capture.

In this study, we aim to evaluate both spatial accuracy and temporal consistency using a unified performance metric. Similar approaches have been proposed in visual object tracking research, where composite evaluation metrics are designed to capture multiple aspects of tracking performance, including accuracy, robustness, and temporal stability [23,24].

In addition, various performance metrics have been developed in the field of computer vision for multi-object tracking (MOT), such as MOTA and MOTP [25], IDF1 [26], and HOTA [27]. These metrics combine detection, localization, and identity association based on Intersection-over-Union (IoU) criteria, but they are primarily suited for evaluating multiple targets. In contrast, optical tracking with a narrow-field telescope focuses on a single target that must remain continuously centered and stable, where angular precision and temporal persistence are dominant. The proposed PWTI therefore aims to integrate these two essential aspects into a unified and physically interpretable measure of tracking quality.

Given these considerations, a different approach to performance evaluation may be required, one that places more emphasis on continuous high-precision tracking and its temporal persistence. This motivates the development of a more targeted metric, which will be introduced and formalized in the following section.

## 3. Tracking Performance Metric and Optimization Framework

### 3.1. System Overview and Target Scenario

The tracking process considered in this study is based on a two-stage optical observation structure: a wide-field search telescope is used to conduct an initial detection and short-arc tracking of a space object, followed by IOD. The resulting estimate is then passed to a narrow-field identification telescope, which must capture high-resolution imagery for classification and identification purposes.

In this context, the initial orbit determination process plays a critical role in handing over the target trajectory from the wide-field to the narrow-field tracking phase. However, IOD based on limited optical observations is inherently sensitive to the timing and spatial characteristics of the measurements used. Unlike radar or laser systems that can directly measure range, optical sensors observe only angular information, making the orbit estimation process more susceptible to divergence or inaccuracy, especially when the observations span a short arc. The quality of the resulting orbit prediction can vary significantly depending on the geometry of the observation, such as the target’s elevation profile at the time of measurement. In particular, when tracking continues toward high-elevation regions based on an IOD result obtained earlier in the ascent, the angular velocity of the target typically increases, and even modest estimation errors can result in significant deviations in the narrow-field tracking phase.

**Figure 1:**
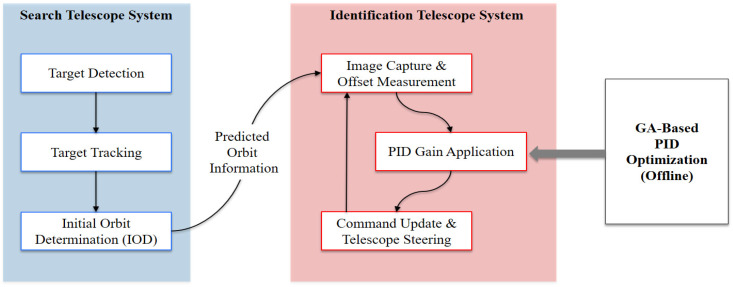
Conceptual Diagram of the Dual-Telescope Tracking System with GA-Based PID Optimization.

Figure 1 illustrates the overall configuration of the dual-telescope tracking system developed in this study. The search telescope performs wide-field detection, short-arc tracking, and initial orbit determination (IOD), whose results are handed over to the identification telescope for narrow-field tracking. Within the identification telescope system, the image-based offset is measured from each captured frame, and correction commands are generated by applying pre-optimized PID gains obtained from a GA-based PID optimization process conducted offline. This framework establishes the operational link between orbit prediction and closed-loop offset correction in the narrow-field phase.

Because the field of view of the identification telescope is extremely narrow, the observed object must remain close to the center of the image for successful high-resolution capture. Tracking is carried out by computing frame-by-frame offset values based on the discrepancy between predicted and actual image locations. These offsets are then translated into correction commands that are applied through the system’s existing control interface.

For simulation and tuning, we constructed a representative set of observation scenarios derived from real satellite trajectories. From this set, we designated a subset of challenging scenarios—typically low orbital altitude with high maximum elevation—as stress-test conditions. These trajectories tend to exhibit increased nonlinearity and rapid variations in both speed and angular velocity, and they are also associated with larger IOD errors due to geometric sensitivity. As such, these cases provide an appropriate benchmark for evaluating whether the tracking system can maintain sustained and precise alignment near the image center under challenging real-world conditions.

### 3.2. Revisiting Previously Used Metrics in the Context of Optical Identification

In earlier studies, we explored PID gain optimization schemes using performance indices such as RMS error and persistence time (PT), applying both grid search [1] and GA techniques [2,3]. These metrics served as practical approximations of tracking performance. RMS quantified the average deviation from the image center, while PT measured how long the system kept the offset within a fixed threshold, such as 5 arcseconds. Although each provided useful insights into controller behavior, they had limitations when applied to tasks that demand sustained, high-precision alignment during optical identification.

One issue with RMS is that it can be overly influenced by transient spikes in error, even if the system subsequently stabilizes well. On the other hand, PT reflects the duration of stable tracking, but treats all offsets within the threshold as equivalent. This means it does not distinguish between precise alignment within 1 arcsecond and looser tracking near 4 arcseconds. Such ambiguity becomes more problematic in high-elevation scenarios, where even brief tracking losses may result in failed imaging or missed identification opportunities.

These limitations suggested the need for an alternative performance index that could more directly reflect the dual requirements of spatial precision and sustained tracking. This led to the development of a new metric, introduced in the following section.

### 3.3. Definition of the Persistence-Weighted Tracking Index (PWTI)

Optical identification systems require sustained and accurate alignment of the target near the center of the field of view. Unlike general tracking applications where short-term accuracy may suffice, optical identification demands both spatial precision and temporal stability, particularly under narrow field-of-view constraints.

Existing performance indicators, such as RMS error and PT, have been used to assess tracking quality, but each has limitations. RMS reflects average deviation but does not account for continuity, while PT captures the duration spent within a given error threshold but treats all in-threshold errors equally, regardless of their proximity to the center.

To better reflect the dual requirement of precision and consistency, we introduce a new metric: the Persistence-Weighted Tracking Index (PWTI). This metric evaluates the quality of continuous tracking segments by scoring each frame according to the magnitude of its positional error and summing the scores over the longest stable segment. Formally, PWTI is defined as:

(1) $$PWTI=\sum_{i\in L_{k}}S\left(e_{i}\right)$$

where $L_{k}$ represents a continuous segment of time indices and $S\left(e_{i}\right)$ is a frame-wise score determined by the absolute tracking error $e_{i}$. The scoring function $S\left(e\right)$ is designed to be linearly decreasing within a practical tolerance range. In this study, the following structure was used:

(2) $$S\left(e\right)=\;\left\{\begin{array}{c} 10\;\;\;if\;0\leq e<1\\ \begin{array}{c} 9\;\;\;\;if\;1\leq e<2\\ \begin{array}{c} 8\;\;\;\;if\;2\leq e<3\\ 7\;\;\;\;if\;3\leq e<4 \end{array}\\ 6\;\;\;\;if\;4\leq e<5 \end{array}\\ 0\;\;\;\;\;\;otherwise \end{array}\right.$$

This design reflects the requirement for high-precision alignment in optical systems and imposes a clear penalty for deviations beyond a certain threshold.

The 5-arcsecond limit was selected based on typical narrow-field imaging systems, where the usable field of view often spans only 10 to 15 arcseconds in width. Beyond this range, the target may drift toward the image boundary, leading to degraded resolution or identification failure. Hence, errors exceeding this practical limit are excluded from scoring.

The stepwise score decay from 10 to 6 reflects a pragmatic balance between tracking precision and robustness. Rather than being mathematically optimized, the scoring scale was empirically tuned to differentiate between near-perfect alignment and marginally acceptable positioning, while avoiding abrupt penalties that might exaggerate the impact of minor deviations. This weighting structure, although empirically chosen, follows the same rationale as conventional composite indicators that combine accuracy and persistence, such as RMS and PT. The intent is to quantify sustained high-precision tracking under practical system constraints rather than to derive an analytically optimal function. The validity of this design was confirmed through simulation-based evaluation, where GA-PWTI optimization consistently produced more stable and precise tracking across all scenarios compared to RMS- and PT-based tuning.

In addition, by computing the score over the longest uninterrupted segment, the metric naturally suppresses the impact of transient noise or isolated outliers. Unlike methods that simply accumulate all in-threshold frames, this structure emphasizes sustained tracking quality and reduces sensitivity to short-term disturbances, improving robustness in realistic tracking conditions.

The resulting PWTI value captures the best stable tracking segment, giving preference to periods with both low error magnitude and temporal continuity. Compared to RMS or PT alone, this index more effectively represents tracking quality in scenarios where accurate and sustained positioning is essential for optical identification.

### 3.4. Genetic Algorithm-Based PID Gain Optimization

To optimize the PID controller parameters under various performance indices, this study adopts a GA framework designed for flexible integration of different performance metrics, including RMS error, persistence time, and the proposed PWTI.

#### 3.4.1. Optimization Framework Overview

Genetic algorithms are population-based, heuristic optimization techniques inspired by biological evolution. They are particularly suitable for control systems with non-convex, high-dimensional, or discontinuous performance surfaces, where gradient-based optimization is ineffective.

In this study, the GA is applied to optimize the three PID gains—Kp, Ki, and Kd—under a unified optimization structure where Kp denotes the proportional gain, Ki the integral gain, and Kd the derivative gain. The goal is to minimize or maximize a performance index PI, which can be flexibly defined depending on the metric in use (e.g., RMS, Persistence Time, or PWTI).

The GA procedure consists of the following stages:

1. Initialization: An initial population of PID gain sets (Kp, Ki, Kd)) is randomly generated within predefined bounds.
2. Evaluation: Each individual (i.e., PID set) is evaluated via simulation using the selected performance index.
3. Selection: Individuals with superior performance are selected as parents.
4. Crossover and Mutation: Genetic operators are applied to generate new offspring, ensuring both exploitation and exploration.
5. Termination: The process iterates over generations until convergence or a maximum number of iterations is reached.

**Figure 2:**
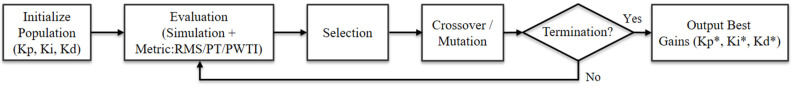
Flowchart of the GA-Based PID Optimization Process.

This modular structure allows direct comparisons across performance metrics using the same optimization engine. The GA optimization is executed entirely offline during the design phase and does not operate in the real-time tracking loop. The overall GA flow is summarized in Figure 2, which visualizes the offline optimization process described above.

#### 3.4.2. Parameter Settings and Simulation Setup

The GA was implemented in MATLAB (R2020b) with a lightweight custom framework. Table 1 summarizes the parameter configuration used across all optimization scenarios. Each PID configuration is evaluated through Monte Carlo simulation with a fixed number of runs to ensure statistical robustness. The resulting performance index is averaged and used as the individual’s fitness value.

#### 3.4.3. Metric-Specific Optimization Strategies

Depending on the selected metric, the GA evaluates PID candidates differently:

- RMS error: Measures the average magnitude of tracking error across the entire sequence.
- Persistence Time: Captures the longest continuous time span during which the tracking error remains below a predefined threshold (e.g., 10 arcsec).
- PWTI (Proposed): Integrates both the size and duration of tracking error into a unified score, giving higher weights to long-lasting small errors and penalizing large or intermittent deviations.

This flexibility in metric definition enables direct comparison of optimized results under identical environmental conditions.

#### 3.4.4. Genetic Optimization Results

**Figure 3:**
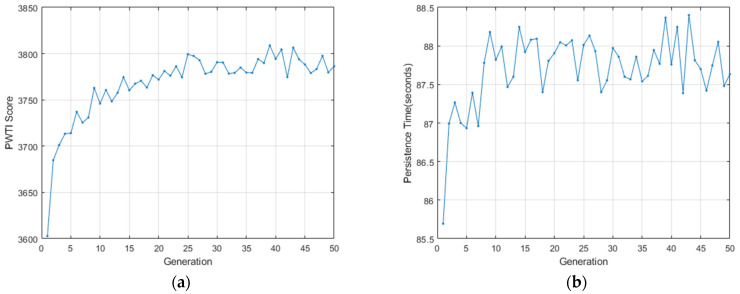
Convergence of genetic algorithm over 50 generations for PWTI-based PID tuning. (**a**) Best PWTI score per generation; (**b**) Corresponding PT values.

To evaluate the effectiveness of the proposed performance metric in a realistic tuning environment, we applied a GA to search for PID gain combinations that maximize the PWTI score. The optimization was conducted over 50 generations with a population size of 30, consistent with prior tuning experiments. The evolution of the best score per generation is shown in Figure 3, where Figure 3a presents the trend of PWTI values and Figure 3b shows the corresponding PT.

In Figure 3a, the best PWTI score shows a clear upward trend in the early generations, followed by gradual stabilization around the 30th generation. This convergence indicates that the GA was able to consistently improve the controller’s ability to maintain precise and temporally stable alignment. The PWTI score increased from approximately 3600 to over 3800, reflecting simultaneous gains in accuracy and tracking continuity.

In parallel, Figure 3b shows that PT improved from 85 s to around 88 s, confirming that the optimized controller not only tracked the target more accurately but also sustained alignment for longer periods. While minor fluctuations are observed in later generations, both metrics reached stable levels, suggesting that the GA had approached a near-optimal region in the solution space.

In terms of gain values, the PID parameters converged toward consistent values across generations, particularly in the proportional and derivative terms. This reinforces the reliability and repeatability of the optimization process under the proposed performance index.

Together, these results demonstrate that the PWTI-based objective successfully guided the genetic search toward effective controller settings for image-based offset tracking. The metric’s ability to incorporate both error magnitude and temporal continuity makes it well suited to the demands of optical identification systems that rely on narrow field-of-view alignment.

## 4. Simulation and Evaluation Results

To evaluate the effectiveness of the proposed performance metric and PID tuning framework, we conducted simulation-based experiments that replicate realistic operational conditions of optical tracking systems. The focus was to assess whether the proposed PWTI enables more accurate and temporally stable tracking performance compared to conventional indicators such as RMS error and PT.

The simulated scenarios represent the identification phase of tracking unknown space objects, where no prior orbital information is available. Each case begins with IOD using a limited set of optical streak images and transitions into a narrow FOV tracking process that relies on image-based offset correction. The goal of this experiment is to validate the practical utility of PWTI and compare it against conventional tuning approaches in a statistically consistent manner.

### 4.1. Simulation Setup and Scenario Design

A total of eleven observation scenarios were constructed by propagating real two-line element (TLE) data of low Earth orbit (LEO) satellites using the Systems Tool Kit (STK 11.5) simulation platform. The satellites used in these simulations were selected from publicly available catalogs of observable objects over the Korean peninsula, including representative cases such as the ISS (≈300 km altitude), WorldView-1 (≈500 km), and KOMPSAT-3 (≈700 km). Each trajectory was generated within STK by considering orbital perturbations and visibility geometry at the observation site, ensuring that the simulated motion closely follows realistic orbital behavior. To reflect diverse tracking conditions, the simulated targets were designed with different maximum elevation angles (e.g., 45°, 60°, 80°), which represent practical variations in visibility and observation geometry. These configurations served as representative test cases for evaluating the proposed control and tuning framework under realistic constraints.

In each scenario, it was assumed that the target had been detected and followed using a wide-field search telescope. Three optical streak images acquired at several-second intervals were used to perform IOD based on angular-only measurements using a conventional Gooding method implemented in the Orbit Determination Tool Kit (ODTK 6.6). The resulting trajectory prediction was transferred to a narrow-field identification system, which performs fine tracking using frame-by-frame offset correction. The control process operates by combining the predicted position from IOD with the accumulated offset from the previous frame to determine the next pointing direction. The observed offset at each step is then used to update subsequent commands. Although this structure resembles a PID-like behavior, it is not a conventional feedback loop with a fixed reference or known system model; rather, it is a simplified correction mechanism based on image error feedback.

The simulation environment was configured to reproduce realistic optical tracking conditions and to examine the effectiveness of the offset-based correction under practical constraints. The frame rate of 5 Hz corresponds to the actual update rate of the identification telescope, providing sufficient temporal resolution while maintaining stable image acquisition. The tracking duration (approximately 100–220 s) was obtained from STK visibility analyses using publicly available TLE data of satellites observable over the Korean peninsula, representing realistic visible-arc intervals for narrow-field ground observations. The effective field of view (±15–20 arcsec) corresponds to the usable region of the telescope detector, beyond which targets are no longer observable. The IOD error applied in the simulation was derived from the actual IOD process described above, rather than from an assumed uncertainty model. The mount dynamics were simplified based on system-identification tests conducted on the real telescope mount, where multiple excitation inputs were applied and the resulting angular responses were analyzed to obtain an equivalent model suitable for simulation.

These parameters collectively ensure that the simulated scenarios accurately reflect real operational conditions and enable a fair evaluation of the proposed PWTI-based tuning framework.

The following four tuning strategies were evaluated across all scenarios:

1. Grid-RMS: PID gains selected through grid search to minimize RMS error.
2. GA-RMS: Genetic algorithm tuning using RMS error as the fitness function.
3. GA-PT: Genetic algorithm tuning using persistence time as the objective.
4. GA-PWTI: Genetic algorithm tuning using the proposed PWTI as the optimization metric.

All configurations were tested with 100 monte carlo runs per scenario. Tracking performance was assessed using three metrics—RMS, PT, and PWTI—and averaged across trials to ensure fair and statistically robust comparison.

### 4.2. Performance Comparison Across Metrics

To quantitatively evaluate the effectiveness of each PID tuning method, three performance metrics were compared across eleven observation scenarios: RMS tracking error, PT, and the proposed PWTI. The results are presented in three separate tables for clarity: Table 2 (RMS), Table 3 (PT), and Table 4 (PWTI).

In this study, each observation scenario was classified into one of two conditions—Nominal or Harsh—based on trajectory geometry. Nominal conditions correspond to relatively smooth trajectories with moderate elevation changes, while Harsh conditions represent challenging cases with low orbital altitude and high maximum elevation, where nonlinear motion and geometric sensitivity are more significant. These classifications are reflected in the “Condition” column of Table 2, Table 3 and Table 4.

Table 2 shows the RMS tracking errors (in arcseconds) for each tuning strategy across all test scenarios. Lower RMS values correspond to higher tracking accuracy, as they represent the average deviation between the predicted and observed target positions. The GA-PWTI method recorded the lowest RMS error in 10 out of 11 cases, often reducing the error by 30–50% compared to the Grid-RMS baseline. GA-RMS and GA-PT also improved over the grid-tuned baseline, but to a lesser degree than GA-PWTI.

One exception was found in the most challenging case (Traj-11), where GA-PWTI exhibited a slightly higher RMS error than the other methods. However, this trade-off is revisited in later sections, where it is shown that GA-PWTI still offers superior performance in temporal metrics even in this case.

The RMS results confirm that PWTI-based tuning is not limited to optimizing its own metric but also leads to reduced average tracking error, likely due to improved trajectory stability.

Table 3 presents the persistence time (PT) in seconds, defined as the longest continuous period during which the tracking error remains within ±5 arcseconds. Higher PT indicates more stable and persistent tracking performance, as it reflects how long the system can continuously stay within the specified error gate. GA-PWTI dramatically outperformed all other methods, often sustaining alignment well beyond 100 s, while other methods remained below 35 s in most cases.

In fact, GA-PWTI achieved PT values that were on average about 5 times longer than GA-PT (typically 4–6 times across trajectories) and up to 53 times longer than the fixed Grid-RMS method. These results validate that the PWTI-based tuning not only minimizes average error but also greatly enhances tracking continuity, which is vital for high-resolution optical identification.

Table 4 summarizes the PWTI scores, which quantify tracking quality by jointly evaluating spatial precision and temporal continuity. Higher PWTI indicates better overall performance, rewarding both small errors and long in-threshold duration. Here, the dominance of GA-PWTI is even more evident. In most cases, its score was 5–7 times higher than GA-PT and 30–62 times higher than Grid-RMS.

For example, in nominal scenarios such as Traj-06, GA-PWTI achieved a PWTI score exceeding 8500, compared to 1290 for GA-PT and only 140 for Grid-RMS. Even in the most challenging scenario (Traj-11), GA-PWTI still maintained a score over 3600, while all other methods fell below 1000.

These results demonstrate that GA-PWTI tuning delivers a significant improvement in the proposed performance metric, while maintaining competitiveness on conventional metrics such as RMS and PT. It consistently provides the most reliable alignment behavior under diverse tracking conditions.

### 4.3. Visual Analysis of Tracking Behavior

To complement the quantitative comparisons presented in Section 4.2, this section provides a visual interpretation of the tracking behavior under different tuning strategies. While the previous section focused on tabulated metrics such as RMS error, persistence time, and the proposed PWTI score, the current section aims to illustrate how those performance differences manifest over time and in the image plane.

**Figure 4:**
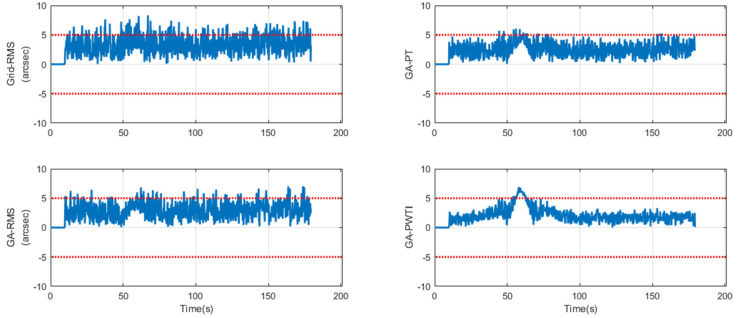
Time-series tracking error by tuning scheme.

Figure 4 shows the frame-by-frame tracking error over time. The *x*-axis represents time in seconds, and the *y*-axis shows the total offset magnitude in arcseconds, computed as the Euclidean norm of the azimuth and elevation errors, $r\left(t\right)=\;\sqrt{{Az}^{2}+{El}^{2}}$. A horizontal dashed line marks the tracking gate at 5 arcseconds; values below this line indicate in-threshold operation. Each panel corresponds to one tuning scheme, and all panels share identical axes to enable direct visual comparison.

In the most challenging scenario, GA-PWTI begins with a slightly larger transient around the handover but settles quickly and then maintains a consistently low error level for the remainder of the track. The curve stays well within the 5 arcsecond gate, with few boundary touches and small local variations.

By contrast, Grid-RMS and GA-RMS enter the gate but exhibit shorter stable stretches and more frequent excursions near or above the threshold, which appears as a more irregular trace. GA-PT achieves longer in-threshold duration than the RMS-oriented schemes, yet its curve runs closer to the 5 arcsecond boundary and shows greater fluctuation, indicating weaker spatial centering despite temporal persistence.

Taken together, these patterns visualize the same advantage captured by the PWTI metric: not only remaining within the threshold, but do so for extended periods with reduced variability.

**Figure 5:**
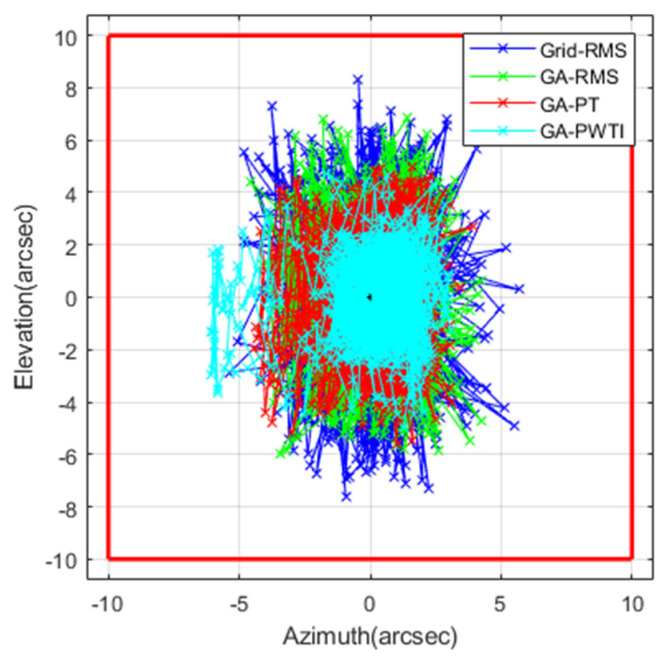
FOV-centered scatter of image-plane offsets.

Figure 5 overlays the accumulated azimuth and elevation offsets for all frames across the four tuning schemes in a single plot centered on the field of view. The *x*-axis shows azimuth error (arcseconds) and the *y*-axis shows elevation error (arcseconds); the origin marks the image center. A red square denotes the ±10-arcsecond reference bounds.

From Grid-RMS through GA-RMS and GA-PT to GA-PWTI, the point cloud progressively contracts toward the origin, indicating improved centering and reduced spread. GA-PWTI produces the tightest concentration near the center. A faint spill in the azimuth direction is visible; this corresponds to the brief departure near peak elevation seen in Figure 4, after which the trajectory returns toward the center.

Overall, the overlaid scatter makes the trend clear: PWTI-based tuning improves both spatial centering and consistency relative to RMS- and PT-oriented baselines.

**Figure 6:**
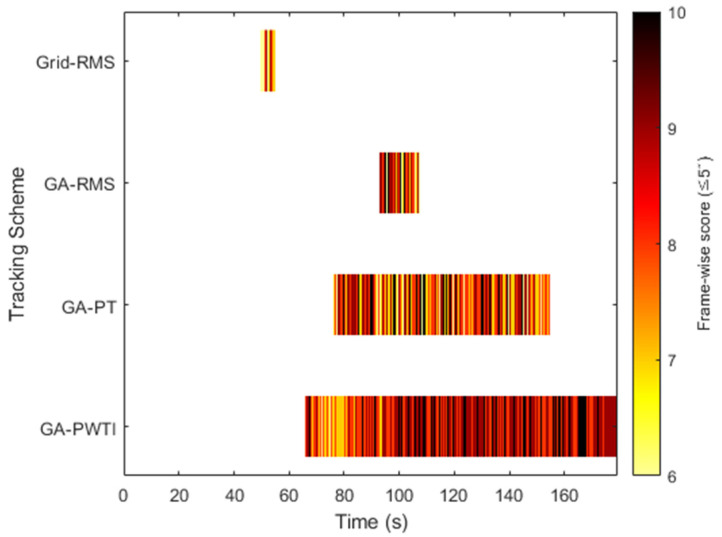
Longest stable in-threshold tracking segments across tuning methods, with color indicating spatial error magnitude.

Figure 6 provides a comparative view of stable tracking performance across the four tuning schemes. For each method, the plot shows the single longest continuous segment during which the tracking error remained below 5 arcseconds. Only this in-threshold segment was extracted from the simulation results for clarity.

A color scale (hot colormap) is applied, accompanied by a colorbar indicating the frame-wise score corresponding to tracking error magnitude. The length of each bar indicates the duration of the stable segment, while the color variation represents spatial accuracy—darker red tones correspond to smaller tracking errors, and brighter yellow tones represent larger offsets from the image center within the allowed threshold.

Among all methods, the GA-PWTI configuration shows the longest and darkest segment, meaning that it maintained the target within the desired error bounds for the longest period and with consistently small errors—demonstrating stable and precise alignment. In contrast, Grid-RMS and GA-RMS yield shorter segments, reflecting limited ability to sustain threshold-level accuracy, while GA-PT achieves longer duration but with lighter coloration, indicating larger offsets in tracking precision.

Overall, this visualization clearly demonstrates what the PWTI metric is designed to reward—not merely staying within an error threshold, but maintaining precise and stable alignment over time. The use of color and the accompanying colorbar enhance the intuitive readability of the results, allowing both temporal stability and spatial precision to be assessed at a glance.

**Figure 7:**
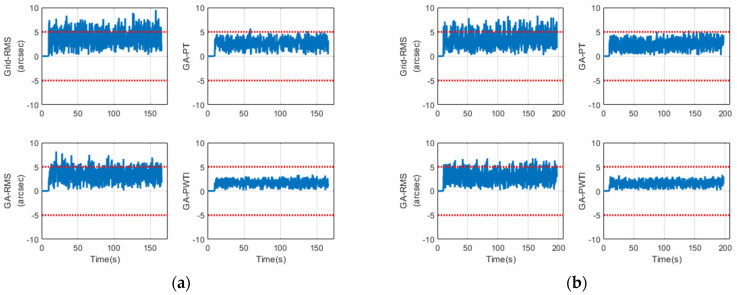

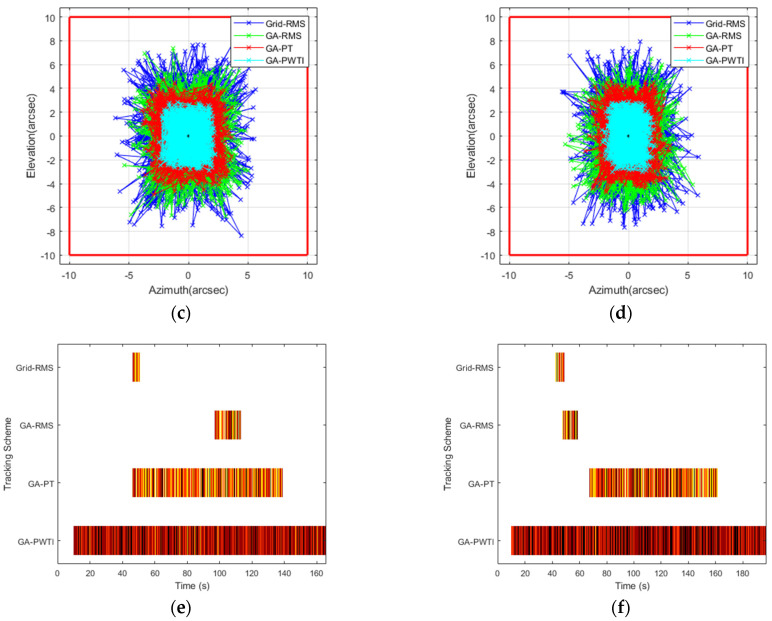
Nominal trajectories: Traj-05 (**left** column) and Traj-08 (**right** column). Rows show, from top to bottom, time-series tracking error, FOV-centered scatter, and the longest in-threshold segment. Panels (**a**,**b**) correspond to time-series, (**c**,**d**) to FOV scatter, and (**e**,**f**) to segments.

Figure 7a–f compare two nominal trajectories—Traj-05 (left) and Traj-08 (right). From top to bottom: time-series error (5-arcsecond gate), FOV-centered scatter (±10-arcsecond bounds), and the longest in-threshold segment per scheme (color shading indicates mean tracking error based on the same hot colormap used in Figure 4); axes and legends are identical across panels.

In both nominal cases, the same trend seen in the challenging scenario becomes even clearer: GA-PWTI stabilizes quickly, remains well within the gate, forms the tightest and most centered cluster, and maintains the longest stable segment. GA-PT shows moderate improvement, while RMS-based methods exhibit shorter and less stable tracking.

These results confirm that the advantages observed under challenging conditions generalize to typical operations—PWTI-based tuning consistently enhances temporal stability, spatial centering, and overall tracking continuity, which are essential for narrow-FOV optical identification.

## 5. Conclusions

This study proposes a new performance metric, the Persistence-Weighted Tracking Index (PWTI), to better reflect the requirements of optical identification systems, where both spatial accuracy and temporal stability are critical under a narrow field of view. Conventional indicators such as RMS error and persistence time each capture only part of tracking quality and do not fully quantify the sustained high precision alignment required for reliable imaging.

By integrating PWTI into a genetic algorithm-based PID tuning framework, we show that the proposed approach outperforms baseline schemes across representative tracking scenarios. In simulation, PWTI tuned controllers achieve lower error and maintain the target near the image center for extended periods, including under the most challenging observational conditions. The advantage is evident in temporal profiles, image plane distributions, and segment level stability.

These findings suggest that performance evaluation metrics designed with application specific constraints in mind, such as sustained alignment for optical imaging, can significantly improve tracking effectiveness. While the present work focuses on simulation-based validation, the modeling and evaluation framework were derived from real observation data and represent practical operating conditions. Nevertheless, further refinement and on-sky testing are required to confirm robustness under diverse sensing environments and system latencies. Future studies will focus on applying the proposed approach to operational optical systems, verifying performance under actual observation conditions, and extending the tuning framework for different imaging missions.

## Figures and Tables

**Table 1:** GA Parameter Settings.

Parameter	Value
Population size	30
Maximum generations	50
Crossover rate	0.8
Mutation rate	0.1
Evaluation method	Depends on PI (RMS/PT/PWTI)
Search range Kp	[0.9–2.0]
Search range Ki	[0.9–2.0]
Search range Kd	[0.00–0.06]

**Table 2:** RMS Comparison by Tuning Scheme for Each Trajectory (100 Monte Carlo Runs). * most challenging high-elevation scenario.

Case	Condition	RMS [arcsec]			
		Grid-RMS	GA-RMS	GA-PT	GA-PWTI
Traj-01	Nominal	4.2914	3.8987	3.0638	1.991
Traj-02	Nominal	4.2936	3.8928	3.0746	1.9974
Traj-03	Nominal	4.204	3.8279	3.007	1.9486
Traj-04	Nominal	4.2758	3.9049	3.0596	1.9873
Traj-05	Nominal	4.2929	3.9002	3.0604	1.9872
Traj-06	Nominal	4.3211	3.9168	3.0811	2.0015
Traj-07	Nominal	4.2901	3.8985	3.0522	1.9836
Traj-08	Nominal	4.3109	3.9228	3.0807	2.0003
Traj-09	Harsh	4.3198	3.9201	3.0882	2.0116
Traj-10	Harsh	4.4087	4.0277	3.301	2.6353
Traj-11	Harsh *	5.9122	5.8314	6.1831	8.1468

**Table 3:** PT Comparison by Tuning Scheme for Each Trajectory (100 Monte Carlo Runs). * most challenging high-elevation scenario.

Case	Condition	PT [s]			
		Grid-RMS	GA-RMS	GA-PT	GA-PWTI
Traj-01	Nominal	3.562	5.4	30.814	161.8
Traj-02	Nominal	3.584	5.354	29.142	165.4
Traj-03	Nominal	3.256	4.972	28.534	98.8
Traj-04	Nominal	3.732	5.392	30.516	151.2
Traj-05	Nominal	3.624	5.32	30.306	156
Traj-06	Nominal	3.782	5.49	33.988	199.8
Traj-07	Nominal	3.662	5.514	28.198	146.4
Traj-08	Nominal	3.538	5.564	33.208	187.4
Traj-09	Harsh	3.842	5.42	31.944	203.2
Traj-10	Harsh	3.802	5.53	28.808	131.96
Traj-11	Harsh *	3.394	4.78	23.478	86.44

**Table 4:** PWTI Comparison by Tuning Scheme for Each Trajectory (100 Monte Carlo Runs). * most challenging high-elevation scenario.

Case	Condition	PWTI			
		Grid-RMS	GA-RMS	GA-PT	GA-PWTI
Traj-01	Nominal	130.96	200.73	1175.01	6943.48
Traj-02	Nominal	132.45	199.74	1109.55	7092.95
Traj-03	Nominal	119.77	185.03	1086.37	4241.09
Traj-04	Nominal	137.01	201.16	1162.18	6489.04
Traj-05	Nominal	134	198.51	1151.45	6696.6
Traj-06	Nominal	140.2	205.67	1293.43	8576.29
Traj-07	Nominal	135.14	205.46	1074.02	6285.71
Traj-08	Nominal	130.37	207.36	1263.11	8045.51
Traj-09	Harsh	141.28	202.02	1214.53	8718.98
Traj-10	Harsh	140.3	205.15	1098.08	5627.41
Traj-11	Harsh *	125.72	177.74	893.73	3670.09

## Data Availability

The data used in this study were obtained from the EOSS project and are not publicly available due to institutional restrictions. No new data were created for this study.

## Abbreviations

The following abbreviations are used in this manuscript:

Az	Azimuth
El	Elevation
FOV	Field of view
GA	Genetic algorithm
GA-PT	Genetic-algorithm tuning using the PT objective
GA-PWTI	Genetic-algorithm tuning using the PWTI objective
GA-RMS	Genetic-algorithm tuning using the RMS objective
IOD	Initial orbit determination
LEO	Low Earth orbit
PID	Proportional–integral–derivative
PT	Persistence time
PWTI	Persistence-Weighted Tracking Index
RMS	Root-mean-square
